# The First Report of Biallelic Missense Mutations in the *SFRP4* Gene Causing Pyle Disease in Two Siblings

**DOI:** 10.3389/fgene.2020.593407

**Published:** 2020-10-23

**Authors:** Anna Sowińska-Seidler, Paweł Sztromwasser, Katarzyna Zawadzka, Dawid Sielski, Ewelina Bukowska-Olech, Paweł Zawadzki, Kazimierz Kozłowski, Aleksander Jamsheer

**Affiliations:** ^1^Department of Medical Genetics, Poznan University of Medical Sciences, Poznan, Poland; ^2^Department of Biostatistics and Translational Medicine, Medical University of Lodz, Łódź, Poland; ^3^MNM Diagnostics, Poznan, Poland; ^4^Molecular Biophysics Division, Faculty of Physics, A. Mickiewicz University, Poznan, Poland; ^5^Department of Medical Imaging, The Children’s Hospital at Westmead, Sydney, NSW, Australia; ^6^Centers for Medical Genetics GENESIS, Poznan, Poland

**Keywords:** Pyle disease, SFRP4, metaphyseal dysplasia, rare disease, WNT signaling, WGS

## Abstract

**Background:**

Pyle disease is a rare autosomal recessive bone dysplasia characterized by the broadening of metaphyses with generalized cortical thinning. Homozygous truncating mutations in secreted frizzled-related protein 4 (*SFRP4*) were, to date, the only known variants causative for this type of skeletal disorder. SFRP4 controls cortical and trabecular bone remodeling by differential regulation of the canonical and non-canonical WNT signaling in both bone compartments. Loss-of-function mutations in the *SFRP4* gene lead to the protein deficiency causing skeletal phenotype typical for Pyle disease.

**Results:**

Herein, we report on the first *SFRP4* missense mutations that occurred in compound heterozygosity in two siblings affected by Pyle disease, and which we have identified using a whole-genome sequencing approach followed by a comprehensive *in silico* pathogenicity assessment. The variants we have found were extremely rare and evaluated to be disease-causing by several online available tools and software.

**Conclusion:**

With this paper, we have shown that Pyle disease may be related not only to *SFRP4* truncating mutations but also to other loss-of-function alterations that possibly impair the protein capacity to bind WNT ligands. As we have expanded here, the range of deleterious variants underlying Pyle disease, we contribute to the knowledge on the pathogenesis of this rare skeletal disorder.

## Introduction

Pyle disease (metaphyseal dysplasia, Pyle type, OMIM: 265900) is an extremely rare autosomal recessive skeletal dysplasia characterized by the widening of metaphyseal trabecular bone with cortical bone thinning. These features affect predominantly long bones being most evident in the distal part of the femur, which shows a characteristic Erlenmeyer flask deformity (Beighton, 1987). The disorder was first described in 1931 by Edwin Pyle in a patient with unusual bone development (Pyle, 1931). One of the most common features of the disease observed in a physical examination is genu valgum, ranging from mild to severe (Beighton, 1987). Humeral bones are usually broadened in their proximal two-thirds, whereas radius and ulna in their distal two-thirds. Other clinical findings include fractures of the metaphysis, bilateral enlargement of the knee, broadening of proximal phalanges and distal metacarpal bones, limitation of elbow extension, dental disorders, mandibular prognathism, joint pain, and muscle weakness. The differential diagnosis includes craniometaphyseal dysplasias and involves the examination of the skull, which is mildly affected in patients presenting with Pyle disease (Beighton, 1987; Gupta et al., 2008).

Recently, homozygous truncating mutations in the *SFRP4* gene (Secreted frizzled-related protein 4; OMIM 606570) have been identified as the genetic cause for the disorder. Kiper et al. (2016) reported the first causative variants in four patients that were two siblings and two unrelated individuals diagnosed with Pyle disease. The disorder was caused by homozygous truncating variants in *SFRP4*: a single nucleotide insertion (c.498_499insG; p.Asp167Glyfs^∗^3) in patients 1 and 2 (the siblings), a nonsense mutation (c.694C>T; p.Arg232^∗^) in patient 3, and a deletion of seven nucleotides (c.481_487delGTACAGG; p.Val161Lysfs^∗^11) in patient 4 (Kiper et al., 2016). In the following year, two other groups reported two additional cases presenting with Pyle disease caused by homozygous nonsense (c.183C>G; p.Tyr61^∗^) and frame-shift (c.315_316delCG; pAsp106Argfs^∗^26) mutations, respectively (Chatron et al., 2017; Galada et al., 2017).

The protein encoded by *SFRP4* belongs to the family of secreted frizzled-related receptors (SFRP1 to 5) that modulate the WNT signaling pathway by binding WNT ligands and thus regulating cell growth and differentiation into specific cell types (Kawano and Kypta, 2003). SFRP4 plays a crucial role in bone remodeling by differential inhibition of the canonical and non-canonical WNT signaling in the cortical and the trabecular bone, hence sFRP-4 deficiency results in a disparate phenotype of both bone compartments (Haraguchi et al., 2016; Kiper et al., 2016; Chen et al., 2019).

Herein, we report on two siblings clinically diagnosed with Pyle disease caused by two novel compound heterozygous missense mutations in the *SFRP4* gene that were detected in both patients using whole-genome sequencing approach (WGS). To our best knowledge, this is the first example of Pyle disease resulting from compound heterozygous missense alterations in *SFRP4*, as all reported causative variants were homozygous truncating mutations.

## Methods

### WGS

Whole-genome sequencing was applied to study a Polish family comprising two affected siblings and their healthy unrelated parents. Genomic DNA was extracted from peripheral blood leukocytes according to standard protocols. The sequencing library was prepared by Macrogen Inc. (Seul, South Korea) using TruSeq DNA PCR-free kit (Illumina Inc., San Diego, California, United States) and 350 bp inserts, and subsequently sequenced on the Illumina Novaseq 6000 platform using 150 bp paired-end reads.

Quality of the sequenced reads was confirmed using FastQC v0.11.7^1^ and the reads were subsequently mapped to GRCh38 human reference genome using Speedseq framework v.0.1.2 (Chiang et al., 2016), BWA MEM 0.7.10 (Li, 2013), Sambamba v0.5.9 (Tarasov et al., 2015). Mapping coverage was calculated using Mosdepth 0.2.4 (Pedersen and Quinlan, 2018). Single nucleotide variants and short indels were detected using DeepVariant 0.8.0 (Poplin et al., 2018). Copy-number variants were called using CNVnator v0.4 (Abyzov et al., 2011). All detected variants were annotated using Ensembl Variant Effect Predictor 97.3 (McLaren et al., 2016).

Detected mutations were referred to online databases of genomic variants including ClinVar^2^ (Landrum et al., 2018), GnomAD^3^ (Karczewski et al., 2020), Human Gene Mutation Database (HGMD) Professional 2014.1^4^ (Stenson et al., 2014), Exome Variant Server (EVS)^5^. The pathogenicity of single nucleotide variants (SNVs) was evaluated *in silico* with the use of multiple online prediction tools including Polyphen-2 (Adzhubei et al., 2010), SIFT (Sim et al., 2012), CADD (Kircher et al., 2014), Mutation Taster (Schwarz et al., 2014), and other resources integrated into VarSome (Kopanos et al., 2019)^6^ and Alamut^®^ Visual^7^ online software products i.e., DANN (Quang et al., 2015), FATHMM (Shihab et al., 2013), LRT (Chun and Fay, 2009), DEOGEN2 (Raimondi et al., 2017), EIGEN (Ionita-Laza et al., 2016), PROVEAN (Choi and Chan, 2015), PhastCons, PhyloP (Siepel et al., 2005; Pollard et al., 2010), and GERP (Cooper et al., 2005). dbNSFP (Liu et al., 2011, 2016) resources were accessed via VarSome. The classification of both SNVs was performed according to the American College of Medical Genetics and Genomics and the Association for Molecular Pathology (ACMG/AMP) (Richards et al., 2015).

### Sanger Sequencing

Targeted Sanger sequencing was performed on DNA samples of all family members subjected to genetic testing to confirm WGS results. Primers for amplification and sequencing were designed for the region of 460 bp using Primer-BLAST software^8^ (Ye et al., 2012). The PCR was performed in a total volume of 25 μl using the containing 5 μl of Q5^®^ Reaction Buffer (New England Biolabs, NEB), 5 μl of Q5^®^ High GC Enhancer (NEB), 1.25 μl of forward (5’ CCTCCTACAAGCCTCAGACG 3’) and reverse primer (5’ ACATGCCCTGGAACATCACG 3’) each (10 μmol/l), 0.5 μl of dNTP mix (10 μmol/l each), 0.25 μl of Q5^®^ High-Fidelity DNA Polymerase (NEB), 2 μl of genomic DNA (50 ng/μl) and 9.75 μl of deionized water. PCR conditions were as follows: initial denaturation at 98°C for 30 s followed by 35 cycles (denaturation at 98°C for 10 s, annealing at 58°C for 20 s, elongation at 72°C for 20 s) and final elongation at 72°C for 5 min. Sequencing of PCR products was carried out using dye-terminator chemistry (kit v.3, ABI 3130XL) and run on automated sequencer ABI Prism 3700 DNA Analyzer (Applied Biosystems).

### Serum Levels of Bone Turnover Markers and Bone Mineral Densitometry

Biochemical analyses of serum levels of total calcium, inorganic phosphate, and vitamin D were performed using standard laboratory methods. The serum level of intact parathyroid hormone was determined with the use of electrochemiluminescence assay (ECL) (Roche, Cobas). The serum calcitonin level was measured using chemiluminescence immunoassay (IMMULITE^®^ 2000 Immunoassay System, Siemens Healthcare). Bone mineral densitometry of the lumbar spine (L1–L4) was performed on the Lunar DPX NT system (GE Healthcare). Results were compared to the laboratory reference ranges.

### 3D Modeling of the SFRP4 Protein Frizzled Domain (FZ)

The 3D structure of the SFRP4 Frizzled domain was predicted using the SWISS-MODEL homology-modeling server^9^ (Waterhouse et al., 2018). As a template, the mouse crystal structure of the cysteine-rich domain of secreted frizzled-related protein 3 (SFRP-3) (ID: 1ijx.1) was used (Dann et al., 2001). The choice of the template was made according to quality criteria, including the homology, global model quality estimate (GMQE), and QMEAN scores (Studer et al., 2020). The 1ijx.1 template had a high sequence identity (73.98%) with SFRP4, an X-ray crystal structure of high resolution (1.9 Å), and a high QMEAN quality score both global (-0.54) and per residue (p.54: 0.87; p.125: 0.86). The modeling was performed for the wild type and both mutated proteins. The obtained model of the wild type SFRP4 protein was further analyzed in mCSM online software^10^ (Pires et al., 2014), which enables to predict the protein stability change upon mutations. The tool uses graph-based signatures to represent protein residue environments by encoding distance patterns between atoms.

## Results

### Clinical Report

**FIGURE 1:**
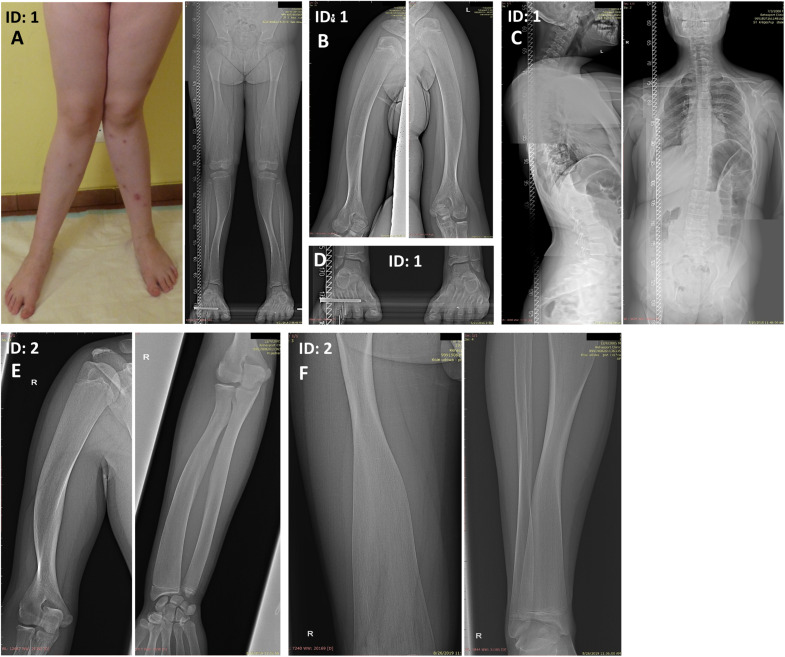
Skeletal malformations observed in the female proband **(A–D)** and her brother **(E,F)**. **(A)** bilateral genua valga with a severe broadening of the knee joints. **(A,F)** Radiographs of the legs showing wide metaphyses and thin cortex of the femoral and tibial bones. **(B,E)** Radiographs of the upper extremities showing broadening of the proximal two-thirds of the humeri and the distal portion of the radial bone. The inward deviation of the distal one-third of the humeri was also noted **(E)**. **(C)** Radiographs of the spine showing thoracolumbar scoliosis. **(D)** Radiograph of the feet showing broadened metatarsals. ID: 1, proband, ID: 2, affected sibling.

The proband (patient 1, ID: 1), a 10-year-old female of Polish ethnicity, was referred to the genetic clinic for a consultation due to suspicion of metabolic bone disorders or skeletal dysplasia. She was born by spontaneous delivery at 38 weeks of gestation after uneventful pregnancy (G3P2) to a healthy non-consanguineous couple, a 27-year-old mother (ID: 3) and 28-year-old father (ID: 4), both of normal height. At birth, the proband’s weight was 3750 g (10th–25th percentile), length 58 cm (97th percentile), head circumference 33 cm (25th–50th percentile), and Apgar score was 9–10. Her psychomotor development was normal. The first symptoms of skeletal disorder occurred at the age of 18 months with the deformation of the tibiae and continued to progress. Upon physical examination performed by the clinical geneticist at the age of 10 years, she was diagnosed with severe Pyle disease manifested by serious skeletal abnormalities, including bilateral genua valga with a severe broadening of the knee joints (Figure 1A), mandibular abnormalities with crowded teeth, and high stature. Radiological examination revealed wide metaphyses and thin cortex of the femur and tibia in both legs (Figure 1A), bilateral broadening of the proximal two-thirds of the humerus (Figure 1B), thoraco-lumbar scoliosis, and broadened metatarsals (Figures 1C,D). The serum levels of bone turnover markers, comprising total calcium, inorganic phosphate, calcitonin, parathyroid hormone, vitamin D concentrations were normal (9.7 mg/dl; 4.3 mg/dl; <2 pg/ml; 57.5 pg/ml; 29.7 ng/ml respectively). Bone mineral densitometry of the lumbar spine (L1–L4) was 0.649 g/cm^2^, which comprise 84% of the normal value for the same age and gender. Bone fractures were not observed.

Patient 2 (ID: 2), the older brother of the proband, was referred for medical examination at the age of 14 years once his WGS results were suggestive for Pyle disease. Although the patient had no signs of genua valga or other overt skeletal deformations, his upper and lower limb X-rays were typical of Pyle disease (Figures 1E,F). The clinical and radiological symptoms were, however, less severe compared to his younger sister. The patient showed a marked broadening of the distal portions of the femoral, tibial, and radial bones. Additionally, proximal parts of the humeral bones were significantly widened, and inward deviation of the distal one-third of the humeri was observed. The patient presented with generalized thinning of the cortical bones (Figures 1E,F).

### WGS and Sanger Sequencing

Whole-genome sequencing of the siblings and their parents yielded 698-1,006M reads resulting in a mean depth of coverage 31.6–45.8X after mapping to the reference genome. Between 96.3 and 98.1% of the genome was covered with at least 20 uniquely mapping reads. A detailed coverage report for all family members is presented in Supplementary Table 1.

**FIGURE 2:**
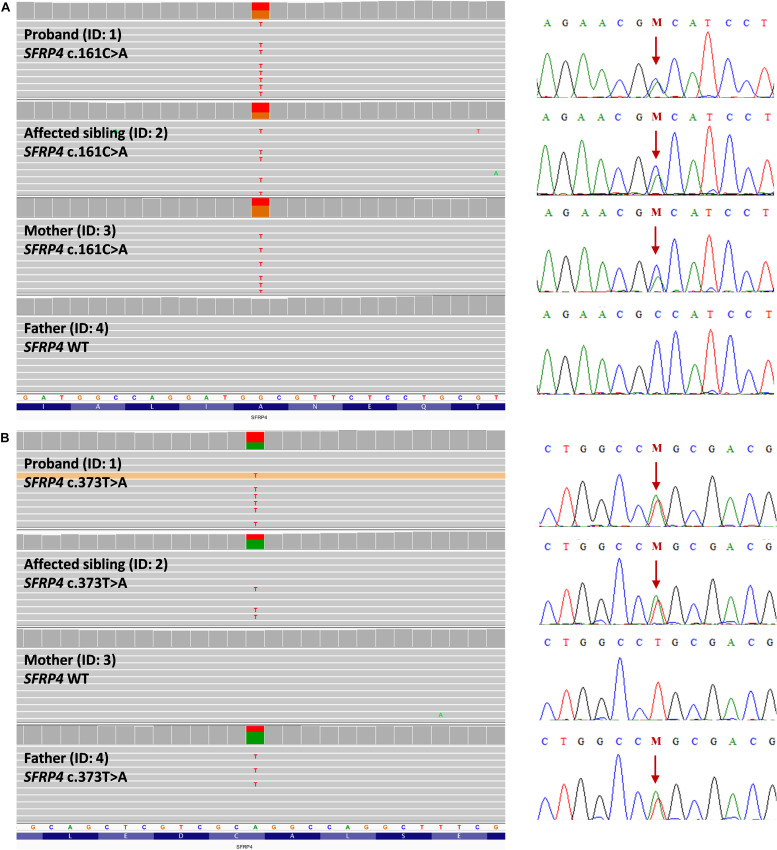
Representation of compound heterozygous *SFRP4* mutations identified in the examined family by means of whole genome sequencing (WGS). The proband (ID: 1) and her brother (ID: 2) were both demonstrated to carry two missense heterozygous variants c.161C>A and c.373T>A. Mutation c.161C>A **(A)** was present in heterozygosity in the unaffected mother, whereas variant c.373T>A **(B)** was shown in heterozygosity in the healthy father. Targeted Sanger sequencing validation of WGS results was represented by corresponding electropherograms for **(A,B)**. Mutations were depicted with “M” and indicated by an arrow.

Using the WGS approach, we have identified compound heterozygous missense mutations NM_003014.4:c.[161C>A];[373T>A] (NP_003005.2: p.[(Ala54Asp)];[(Cys125Ser)]) in exon 1 of the *SFRP4* gene in both affected patients. Segregation studies showed that the unaffected mother was a carrier of variant c.161C>A, whereas variant c.373T>A was inherited from the unaffected father (Figures 2A,B). Both variants were covered with at least 32 reads in all tested samples. Detailed coverage metrics for the *SFRP4* gene are presented in Supplementary Table 2. The WGS results were confirmed by means of targeted Sanger sequencing in all family members subjected to genetic testing (Figures 2A,B).

Both variants are extremely rare. The minor allele frequency (MAF) for variants c.[161C>A] (rs758308395) and c.[373T>A] (rs1344808304) were 0.000008 and 0.000004, respectively (GnomAD v2.1.1). Furthermore, no homozygotes were reported according to GnomAD v2.1.1. Both variants were absent in GnomAD v3, EVS, and 1000 Genomes databases^11^ (accessed August 8, 2020; Table 1). According to our in-house data, variants were not present in 111 Polish controls.

**TABLE 1:** The overview of variants found in the *SFRP4* gene available from online tools.

	c.161C>A p.(Ala54Asp)	c.373T>A p.(Cys125Ser)
gDNA level	Chr7(GRCh38):g.379 16377G>T	Chr7(GRCh38):g.379 16165A>T
dbSNP rs number	rs758308395	rs1344808304
Exon	1	1
Protein domain	Frizzled domain	Frizzled domain
gnomAD (v2.1.1)	ALL: 0.00080%, NFE: 0.0018%	ALL: 0.00040%, NFE: 0.00090%
gnomAD (v3)	–	–
SIFT (v6.2.0)	Deleterious (score: 0)	Deleterious (score: 0)
PolyPhen-2 (v2)	Probably damaging (score: 1)	Probably damaging (score: 1)
CADD Phred	Deleterious (score: 35)	Deleterious (score: 34)
DANN (v2014)	Damaging (score: 0.9971)	Damaging (0.9946)
FATHMM (dbNSFP v4.0)	Damaging (score: -1.55)	Damaging (score: -3.22)
LRT (dbNSFP v4.0)	Damaging (score: 0)	Deleterious (score: 0)
DEOGEN2 (dbNSFP v4.0)	Damaging (score: 0.9279)	Damaging (score: 0.9335)
EIGEN (dbNSFP v4.0)	Pathogenic (score: 0.995)	Pathogenic (score: 0.8743)
MutationTaster (v2013)	Disease causing (accuracy: 1)	Disease causing (accuracy: 1)
PROVEAN (dbNSFP v4.0)	Damaging (score: -5.3)	Damaging (score: -8.74)
PhastCons100way (dbNSFP v4.0)	Conserved (score: 1)	Conserved (score: 1)
PhyloP100way (dbNSFP v4.0)	Conserved (score: 9. 936)	Conserved (score: 7.433)
GERP (v2010)	Conserved (score: 4.6199)	Conserved (score: 4.28)
mCSM: Protein stability Change (ΔΔG)	Highly destabilizing (-2.641 Kcal/mol)	Highly destabilizing (-2.412 Kcal/mol)

**Variants are designated according to the SFRP4 reference transcript NM_003014.4 and protein NP_003005.2. Online tools used: Alamut^®^ Visual software, Varsome, mCSM. gDNA: genomic DNA.**

**FIGURE 3:**
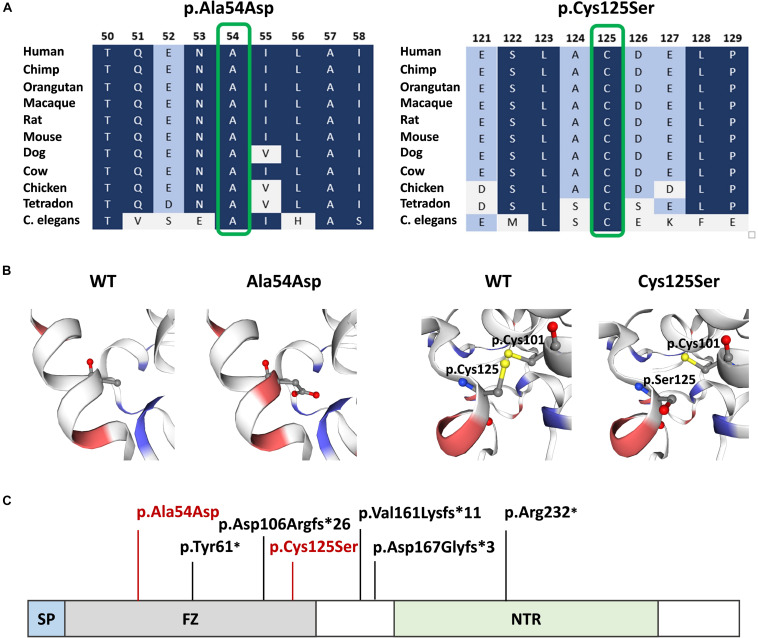
SFRP4 protein structure and conservation. **(A)** Multiple sequence alignment of SFRP4 protein in 11 species available from Alamut^®^ Visual software. Variants p.Ala54Asp and p.Cys125Ser are shown in green boxes. Residues with high conservation among species are shown in navy blue, whereas with lower conservation in blue and gray. **(B)** 3D prediction of p.Ala54Asp and p.Cys125Ser mutation sites and the corresponding WT loci. Red and blue colors indicate negatively and positively charged amino acids. Note the change of charge at position p.54 from non-polar—alanine to negatively charged—aspartic acid. Sulfhydryl side chains of cysteines at positions p.125 and p.101 are indicated in yellow. Disulfide bond is represented by a yellow bar. Note the absence of the disulfide bond in the mutated (p.Cys125Ser) protein. **(C)** Schematic representation of SFRP4 protein. Previously reported mutations are marked in black. The mutations identified in this study are marked in red. SP, signal peptide; FZ, frizzled domain; NTR, netrin-like domain.

Both mutations were evaluated to be deleterious/pathogenic by all tested online *in silico* prediction tools, and both are located in the highly conserved sequence, which was established by GERP (Cooper et al., 2005), PhyloP, and PhastCons (Siepel et al., 2005; Pollard et al., 2010). The pathogenicity and conservation scores for both variants ale listed in Table 1. Multiple sequence alignment of SFRP4 protein available from Alamut^®^ Visual software is represented in Figure 3A.

Variants were neither reported in HGMD, nor ClinVar mutation databases (accessed August 8, 2020). According to ACMG/AMP, both variants were evaluated as likely pathogenic (PM1, PM2, PM3, PP3) (Richards et al., 2015).

### 3D Modeling of the SFRP4 Protein FZ Domain

Graphical presentation of the p.(Ala54Asp) mutation site shows a change in polarity of the amino acid side chains from non-polar—alanine to polar, negatively charged—aspartic acid. 3D analysis of mutation p.(Cys125Ser) shows the change from sulfanyl group-containing cysteine to serine, which contains a hydroxyl group. This substitution results in the absence of a disulfide bond between amino acids at positions p.125 and p.101, which is normally formed in the native protein (Figure 3B). The prediction of the protein stability change employing mCSM tool revealed that both mutations were evaluated as highly destabilizing (Table 1).

## Discussion

The genetic cause of Pyle disease remained unknown for a long time once it was first reported in 1931 (Pyle, 1931). Recently, Kiper et al. (2016) described truncating homozygous mutations in *SFRP4* in three unrelated families of Turkish, Japanese, and Indian ethnicity. The researchers showed using the *sfrp4*-null mouse model, that widening of metaphyseal trabecular bone and cortical thinning, typical for Pyle disease, is referred to differential modulation of non-canonical Wnt signaling pathway in both compartments of the bone. The disruption of *sfrp4* leads to the activation of canonical and non-canonical Wnt signaling in the cortical bone. In contrast, in the trabecular compartment, only the canonical Wnt cascade remains active. Such dysregulation results in decreased periosteal bone formation, increased endocortical bone resorption, and endosteal bone remodeling in the cortex leading to cortical thinning. Conversely, the predominant activation of the canonical Wnt signaling pathway in the trabecular bone results in increased formation of trabecular bone mass. The authors showed that sFRP-4 differentially downregulates the canonical and non-canonical Wnt signaling in both compartments of the bone, allowing for the proper bone thickness and strength (Haraguchi et al., 2016; Kiper et al., 2016; Chen et al., 2019).

The SFRP4 protein is composed of three structural units, i.e., the N-terminal signal peptide (SP), the cysteine-rich domain (FZ) homologous to CDC of the Frizzled receptors and located at the N-terminal part of the protein, and netrin-like domain (NTR), that shares similarity with the axon-guidance protein netrin, located at the C-terminal half of the molecule (Figure 3C). The FZ domain binds WNT ligands, while the NTR unit is crucial for optimal WNT antagonist function (Kawano and Kypta, 2003; Bhat et al., 2007). The homozygous truncating mutations in *SFRP4* identified by Kiper et al. (2016) were located in exons 2 and 4, whereas mutations described by Chatron et al. (2017) and Galada et al. (2017) were both found in exon 1. All variants were predicted to result in either nonsense-mediated mRNA decay or the synthesis of the truncated protein lacking the NTR domain and, thus, most probably affected WNTs inhibition. Additionally, the mutation reported by Galada (p.Tyr61^∗^) was predicted to cause SFRP4 truncation at the FZ domain, probably disabling the protein to bind WNT ligands. Consistent with these findings, it has been suggested that Pyle disease results from loss-of-function mutations in *SFRP4* of which, only the truncating variants have been reported (Kiper et al., 2016; Chatron et al., 2017; Galada et al., 2017).

Interestingly, the mutations identified in our study were missense alterations detected in the compound heterozygous state. Since both variants locate in the exon 1 of the *SFRP4* gene, they affect the FZ domain of the encoded protein. The variants were considered to be deleterious, as both are extremely rare, localize in the evolutionary conserved sequences, and change the physicochemical properties of amino acids. Noteworthy, the substitution of alanine with aspartic acid at position p.54 shifts the amino acid characteristics from non-polar, frequently clustered to the inside of a protein, to polar, negatively charged, hydrophilic, and nearly always found on the outside of a protein structure. Notably, the substitution of cysteine with serine at position 125 prevents the formation of a disulfide bond that is formed in the native protein between paired cysteines at positions p.125 and p.101. Hence, protein folding in the mutant molecule is likely affected. Additionally, the *in silico* prediction of protein stability change classified the two variants as highly destabilizing. Consistent with these findings, both mutations are predicted to impair the ability of SFRP4 FZ domain to bind WNT ligands. However, functional studies are needed to confirm the reduced binding capacity of the mutated protein.

To conclude, herein, we report on the first *SFRP4* missense mutations in compound heterozygosity resulting in Pyle disease. This study supports the discovery that *SFRP4* loss-of-function mutations are causative for this condition, confirming the role of *SFRP4* in bone remodeling. Our findings give a new insight into the pathogenesis of Pyle disease by broadening the spectrum of pathogenic mutations underlying this rare skeletal disorder. The comprehensive understanding of the SFRP4-mediated bone developmental process could contribute to future therapeutic approaches of the diseases associated with either reduced or increased bone mineral density, facilitating, for instance, treatment of osteoporosis and osteopetrosis.

## Data Availability Statement

The datasets for this article are not publicly available due to concerns regarding participant/patient anonymity. Requests to access the datasets should be directed to the corresponding author.

## Ethics Statement

The studies involving human participants were reviewed and approved by the Institutional Review Board of the Poznan University of Medical Sciences. Written informed consent to participate in this study was provided by the participants’ legal guardian/next of kin. Written informed consent was obtained from the individual(s), and minor(s)’ legal guardian/next of kin, for the publication of any potentially identifiable images or data included in this article.

## Author Contributions

AJ recruited and clinically diagnosed the patients, critically revised the manuscript, and supervised the study. KK consulted the proband. AS-S, PS, KZ, DS, and EB-O analyzed data. AS-S drafted the manuscript. PS described WGS approach. PZ provided financial support for the research. All authors contributed to the article and approved the submitted version.

## Conflict of Interest

The authors declare that the research was conducted in the absence of any commercial or financial relationships that could be construed as a potential conflict of interest.

## Footnotes

1 https://www.bioinformatics.babraham.ac.uk/projects/fastqc/

2 https://www.ncbi.nlm.nih.gov/clinvar/

3 https://gnomad.broadinstitute.org/

4 http://www.hgmd.cf.ac.uk/ac/index.php

5 https://evs.gs.washington.edu/EVS/

6 https://varsome.com/

7 https://www.interactive-biosoftware.com/alamut-visual/

8 https://www.ncbi.nlm.nih.gov/tools/primer-blast/

9 https://swissmodel.expasy.org/

10 http://biosig.unimelb.edu.au/mcsm/

11 https://www.internationalgenome.org/
