# Supplementary material for: The First Report of Biallelic Missense Mutations in the SFRP4 Gene Causing Pyle Disease in Two Siblings
Source: Front Genet. 2020 Oct 23;11:593407. doi: 10.3389/fgene.2020.593407 (PMC7646522; doi:10.3389/fgene.2020.593407)
Supplement: Supplementary file 1 [file Data_Sheet_1.docx]

Supplementary Table 1. Coverage metrics and variant counts in the tested samples

| **Patient’s ID** | **Total reads** | **Mean depth (X)** | **Sequence ≥ 10X (%)** | **Sequence ≥ 20X (%)** | **Sequence ≥ 30X (%)** | **Total SNVs** | **Total indels** |
| --- | --- | --- | --- | --- | --- | --- | --- |
| **1** | 1 006 491 878 | 45.8 | 98.5 | 98.1 | 96.7 | 3 912 330 | 967 593 |
| **2** | 993 002 180 | 44.2 | 99.1 | 97.5 | 92.8 | 3 864 517 | 956 211 |
| **3** | 698 054 668 | 31.6 | 98.3 | 96.3 | 70.3 | 3 912 612 | 952 387 |
| **4** | 969 695 522 | 43.3 | 99.1 | 97.3 | 92.4 | 3 860 731 | 951 731 |

Abbreviations: SNVs, single nucleotide variants; indels, insertions and/or deletions

Supplementary Table 2. Coverage details for the *SFRP4* gene in the tested samples

| **Patient’s ID** | **Mean depth (X)** | **Sequence ≥ 10X (%)** | **Sequence ≥ 20X (%)** | **Sequence ≥ 30X (%)** | **c.[161C>A]**  **coverage (X)** | **c.[373T>A] coverage (X)** |
| --- | --- | --- | --- | --- | --- | --- |
| **1** | 48.73 | 100 | 100 | 97.68 | 52 | 47 |
| **2** | 49.73 | 100 | 100 | 99.56 | 48 | 43 |
| **3** | 32.05 | 100 | 97.68 | 67.85 | 36 | 34 |
| **4** | 43.75 | 100 | 100 | 99.16 | 32 | 42 |

Note: Variants are designated according to the *SFRP4* reference transcript NM_003014.4
